# A non-BRICHOS surfactant protein c mutation disrupts epithelial cell function and intercellular signaling

**DOI:** 10.1186/1471-2121-11-88

**Published:** 2010-11-20

**Authors:** Markus Woischnik, Christiane Sparr, Sunčana Kern, Tobias Thurm, Andreas Hector, Dominik Hartl, Gerhard Liebisch, Surafel Mulugeta, Michael F Beers, Gerd Schmitz, Matthias Griese

**Affiliations:** 1 Department of Pneumology, Dr. von Hauner Children's Hospital, Ludwig-Maximilians University, Lindwurmstr. 4, Munich, 80337, Germany; 2 Institute for Clinical Chemistry and Laboratory Medicine, University of Regensburg, Franz-Josef-Strauss-Allee 11, Regensburg, 93053, Germany; 3 Pulmonary and Critical Care Division, University of Pennsylvania School of Medicine, 380 S. University Avenue, Philadelphia, PA 19104, USA

## Abstract

**Background:**

Heterozygous mutations of *SFTPC*, the gene encoding surfactant protein C (SP-C), cause sporadic and familial interstitial lung disease (ILD) in children and adults. The most frequent *SFTPC *mutation in ILD patients leads to a threonine for isoleucine substitution at position 73 (I73T) of the SP-C preprotein (proSP-C), however little is known about the cellular consequences of SP-C^I73T ^expression.

**Results:**

To address this, we stably expressed SP-C^I73T ^in cultured MLE-12 alveolar epithelial cells. This resulted in increased intracellular accumulation of proSP-C processing intermediates, which matched proSP-C species recovered in bronchial lavage fluid from patients with this mutation. Exposure of SP-C^I73T ^cells to drugs currently used empirically in ILD therapy, cyclophosphamide, azathioprine, hydroxychloroquine or methylprednisolone, enhanced expression of the chaperones HSP90, HSP70, calreticulin and calnexin. SP-C^I73T ^mutants had decreased intracellular phosphatidylcholine level (PC) and increased lyso-PC level without appreciable changes of other phospholipids. Treatment with methylprednisolone or hydroxychloroquine partially restored these lipid alterations. Furthermore, SP-C^I73T ^cells secreted into the medium soluble factors that modulated surface expression of CCR2 or CXCR1 receptors on CD4+ lymphocytes and neutrophils, suggesting a direct paracrine influence of SP-C^I73T ^on neighboring cells in the alveolar space.

**Conclusion:**

We show that I73T mutation leads to impaired processing of proSP-C in alveolar type II cells, alters their stress tolerance and surfactant lipid composition, and activates cells of the immune system. In addition, we show that some of the mentioned cellular aspects behind the disease can be modulated by application of pharmaceutical drugs commonly applied in the ILD therapy.

## Background

Biophysically active pulmonary surfactant contains a mixture of lipids and hydrophobic surfactant proteins B (SP-B) and C (SP-C). A normal composition and homeostasis of pulmonary surfactant is critical for its surface-tension-reducing properties and gas exchange in the alveolar region of the lung. SP-C is synthesized exclusively by alveolar type II cells (AECII) as a 21 kDa preprotein (proSP-C). ProSP-C is further processed to the 4.2 kDa mature protein through a sequence of proteolytic cleavages before being secreted together with lipids and other surfactant components to the alveolar surface [1-4]. Surfactant secretion is accomplished by fusion of lamellar bodies, AECII-specific organelles for surfactant assembly and storage, with the plasma membrane [5]. SNARE proteins, in particular syntaxin 2 and SNAP-23, are required for regulated surfactant secretion. Both proteins are associated with the plasma membrane and to some degree with lamellar bodies [6,7]. In parallel to secretion, AECII reinternalize and recycle surfactant components from the alveolar surface by means of endocytosis via clathrin-dependent and clathrin-independent pathways, which include routing to early endosomes and multivesicular bodies [5].

Interstitial lung disease (ILD) is a heterogeneous group of diseases of known and unknown etiology [8-10]. Several histological and clinical subtypes of ILD are linked to the SP-C protein deficiency caused by mutations of the corresponding *SFTPC *gene [3]. Many SP-C mutations cluster within the preprotein's BRICHOS domain and lead to misfolding of the preprotein, aberrant trafficking and processing [11]. To date, all affected individuals with BRICHOS domain mutations have been heterozygous with no detectable mature SP-C in their lungs, suggesting a dominant-negative effect of the mutant allele (e.g. Δexon4, L188Q) [11,12]. Moreover, in cell lines expressing BRICHOS domain mutations, proSP-C forms perinuclear aggregates, consistent with the cell's inability to clear aggregated misfolded proteins and a toxic gain-of-function [12,13]. Various pathologic mechanisms for these mutations causing chronic accumulation of misfolded proSP-C have been proposed, such as induction of endoplasmic reticulum (ER) stress, cytotoxicity, and caspase 3- and caspase 4-mediated apoptosis [14-16]. These factors might contribute to ILD through cell injury and death of AECII. In addition to the BRICHOS domain mutations, a second class of *SFTPC *mutations has emerged. A heterozygous missense mutation, leading to a substitution of threonine for isoleucine at position 73 of the proSP-C, is the most frequent *SFTPC *mutation [17,18]. There is a strong variability in the phenotype of these patients, ranging from asymptomatic to early fatal cases [19]. I73T SP-C (SP-C^I73T^) is marked by mistrafficking of the preprotein to the endosomal compartment and by preserved secretion of both mature and aberrant proSP-C and proSP-B forms and their intra-alveolar accumulation [17,20,21]. Yet, current knowledge on SP-C^I73T ^lacks a precise understanding of the proSP-C processing abnormalities, concurrent cell stress response and cytotoxicity, as well as perturbations of the surfactant composition and secretion.

Current treatment of the genetic interstitial lung diseases in children is unfortunately empirical. Corticosteroids are anti-inflammatory and stimulate surfactant protein transcription [22,23]. Chloroquine and its less toxic derivative hydroxychloroquine [24,25] are used and believed to act on the lysosomal function, i.e. reduce vesicle fusion, exocytosis and proteolytic degradation [26,27] or stimulate lamellar body biogenesis [28]. Thus, there is a need to define the cellular mechanism of the currently applied treatments. Potential therapeutic targets are cell chaperones which assist in normal protein folding and are part of the cytoprotective mechanism that keeps control over misfolded proteins either by folding them correctly or by directing them to the degradation pathway [29,30]. Δexon4 *SFTPC *mutation and proSP-C^Δexon4 ^accumulation upregulate the major ER chaperone BiP in an attempt to maintain surfactant biosynthesis in the presence of ER stress [14]. The regulation of other chaperones, like HSP90, HSP70, calreticulin and calnexin, is unknown. Even so, without pharmacological manipulation, such cytoprotective mechanisms may not be sufficient to maintain production of the bioactive surfactant with a normal lipid/protein composition. In addition, AECII, stressed by aberrantly processed proteins, might signal to and activate the surrounding cells, particularly those of immune system, which could contribute to the SP-C-associated disease.

The goal of this study was to investigate the intracellular disturbances and intercellular signaling of AECII affected by SP-C^I73T ^expression and the ability of the pharmaceuticals commonly used in ILD therapy to modulate some of the cellular mechanisms behind the diseases. We demonstrate the impact of I73T mutation on proSP-C processing, AECII stress tolerance, surfactant lipid composition and activation of the cells of the immune system. In addition, we investigate modulation of the disease cellular mechanisms by pharmaceutical drugs applied in the ILD therapy.

## Results

### MLE-12 cells process proSP-C^I73T ^differently from proSP-C^WT ^and accumulate proSP-C^I73T ^processing intermediates

**Figure 1:**
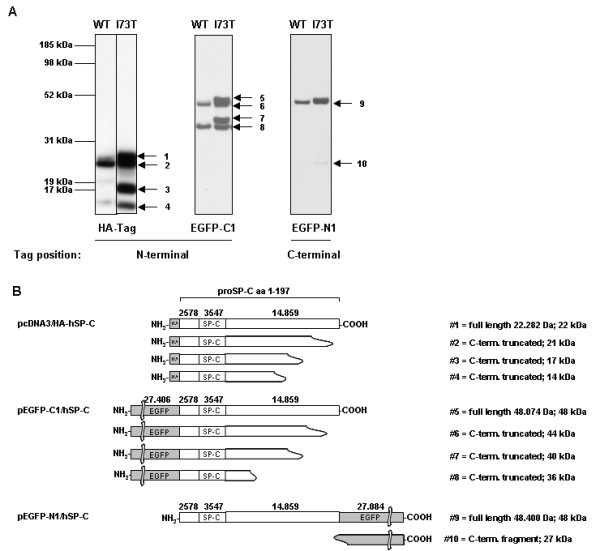
**Processing features of proSP-C^WT ^or proSP-C^I73T^**. (A) Immunoblotting of total cell lysates with tag-specific antibodies. Cell lysates obtained from MLE-12 cells stably expressing fusion protein of proSP-C with an N-terminal HA-tag (left panel), transiently transfected cells expressing fusion protein of proSP-C with an N-terminal EGFP (EGFP-C1, middle panel) or a C-terminal EGFP (EGFP-N1, right panel), present with several bands corresponding to different proSP-C processing intermediates, in which the tag sequence is retained. (B) Based on the size of the bands, the projected corresponding intermediate species of the fusion constructs are depicted. The cleavage sites are only estimates due to the limited resolution of the technique. EGFP-C1 (band #5) and EGFP-N1 (band #9) are expressed as a full-length product of 48 kDa, HA-SP-C (band #1) of size 22 kDa.

SP-C is synthesized exclusively by AECII as a 21 kDa proSP-C which is processed to the 4.2 kDa mature protein through a sequence of C-terminal and N-terminal proteolytic cleavages [2,3]. To identify potential processing differences between proSP-C^WT ^and proSP-C^I73T^, MLE-12 cells were transfected with plasmid vectors, allowing expression of fusion proteins of proSP-C with either C-terminal (N1) or N-terminal (C1) EGFP tag or N-terminal HA-tag. Stable expression of the N-terminally HA-tagged proSP-C^WT ^resulted in appearance of a strong protein band at ~21 kDa and weak bands at 22 kDa, 17 kDa, and 14 kDa (Figure 1A, left). ProSP-C^I73T ^yielded the same four bands, however all at equal intensity in relation to each other, indicating accumulation of proSP-C^I73T ^forms (Figure 1A, left). The postulated processing products based on their size and the fact that the N-terminal HA-tag was still present are depicted in Figure 1B. Mature SP-C was never detectable because of the loss of the protein tag due to the final processing steps at the N-terminus.

Transient expression of N-terminal and C-terminal EGFP fusion proteins with proSP-C were detectable 24 hours post transfection (Figure 1A, middle and right). Again, the processing intermediates of the N-terminally tagged fusion proteins differed between proSP-C^WT ^and proSP-C^I73T^, showing accumulation of all four proSP-C^I73T ^bands for the mutant protein (Figure 1A, middle). In contrast, no differences in the band pattern between proSP-C^WT ^and proSP-C^I73T ^were observed for processing intermediates containing the C-terminal EGFP-tag (Figure 1A, right). This suggests that there is no change in the cleavage pattern regarding the truncation of proSP-C from the C-terminus, being the first proSP-C cleavage step [2]. The lowest band (Figure 1A and 1B, #10) corresponded to the EGFP-tag, which has a size of ~27 kDa.

In summary, the expression of SP-C^I73T ^in MLE-12 cells resulted in the intracellular accumulation of intermediate processing products. Such processing forms are also found in the BAL fluid of patients with this mutation [17,21,31] and may reflect alterations in folding, trafficking and/or processing of proSP-C^I73T^. Based on these initial experiments we considered this *in vitro *cellular system to be an appropriate model to study the effects of *SFTPC *mutations on cellular physiology and stress responses.

### ProSP-C^I73T ^localizes to different intracellular compartments than proSP-C^WT^

**Figure 2:**
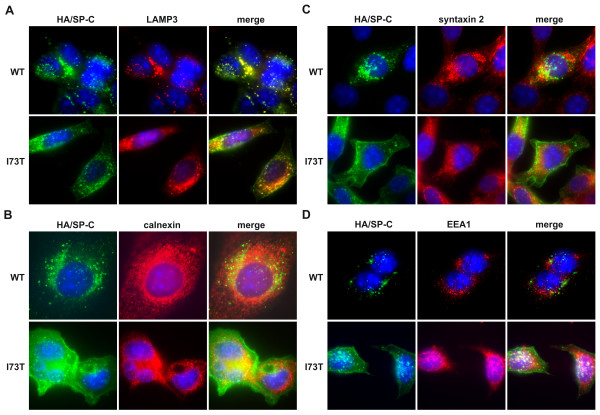
**Intracellular localization of proSP-C^WT ^and proSP-C^I73T ^forms in stabile MLE-12 cells**. Immunofluorescent analysis with an antibody against (A) lamellar body/lysosomal protein LAMP3 (red) and (B) ER protein calnexin (red) show complete localization of proSP-C^WT ^(green) forms and partial localization of proSP-C^I73T ^(green) inside of lamellar body like organelles, and no colocalization of WT or mutant forms with calnexin indicating no ER retention. While proSP-C^WT ^localized with (C) syntaxin 2, a protein found within lamellar bodies as surfactant secretory vesicles, and proSP-C^I73T ^only partially, (D) proSP-C^I73T ^was detectable in a significant amount in EEA1-positive early endosomes. Nuclei are stained with DAPI (blue). Scale bars - 10 μm.

The intracellular localization of preprotein species, monitored by immunofluorescence, differed between proSP-C^WT ^and proSP-C^I73T ^fusion proteins in MLE-12 cells stably expressing N-terminally HA-tagged SP-C. Again, with this approach mature SP-C was not detected because of the loss of the HA-tag due to the final processing steps at the N-terminus with only proSP-C intermediates observed. ProSP-C^WT ^forms were found in the lamellar body-like structures detectable as LAMP3-positive vesicles in MLE-12 cells (Figure 2A). On the other hand, the proSP-C^I73T ^signal was less vesicular with a stronger cytoplasmic background and a pronounced signal at the cell border, but still partially colocalized with the LAMP3 (Figure 2A). This indicates that proSP-C^I73T ^intermediates do traffic to some extent to LAMP3-positive vesicles. None of the proSP-C forms, WT or I73T, colocalized with the ER-specific protein calnexin, suggesting that no proSP-C species were ER retained (Figure 2B). Surfactant secretion is dependent on the fusion of lamellar bodies with the plasma membrane, which requires the activity of SNARE proteins, such as syntaxin 2 and SNAP-23, both associated to some degree with lamellar bodies [6,7]. While proSP-C^WT ^forms colocalized well with syntaxin 2, proSP-C^I73T ^did not (Figure 2C). In contrast, proSP-C^I73T ^intermediates were found partially in early endosomes detected as EEA1-positive vesicles, while proSP-C^WT ^was almost not present in those compartments (Figure 2D), confirming earlier data [17]. Early endosomes usually contain endocytosed material that is destined for recycling or degradation [32]. This suggests that physiological proSP-C^WT ^forms are secreted via lamellar body fusion with the plasma membrane, while some proSP-C^I73T ^forms might take a different route.

### Expression of SP-C^I73T ^increases susceptibility of MLE-12 cells to exogenous stress imposed by pharmacological substances

**Figure 3:**
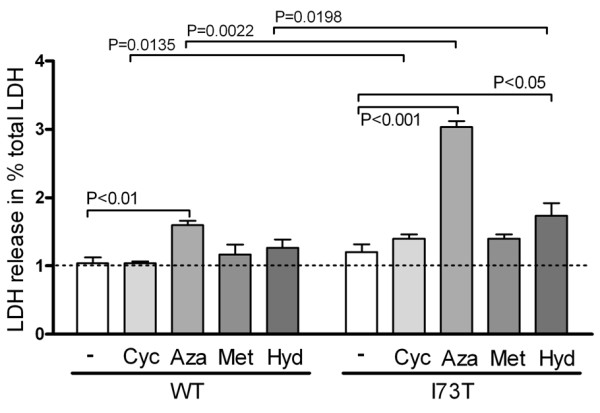
**Viability of MLE12 cells expressing SP-C^WT ^or SP-C^I73T ^before and after treatment with drugs used in therapy**. MLE-12 cells stably expressing SP-C^WT ^or SP-C^I73T ^were incubated 24 hours with 10 μM of cyclophosphamide (+Cyc), azathioprine (+Aza), methylprednisolone (+Met), or hydroxychloroquine (+Hyd). LDH release, a sign of deceased cell fitness, of treated vs. untreated (-) cells is expressed as % of total LDH. Only significant p-values are depicted.

In order to determine the impairment of cells that express SP-C^I73T^, lactate dehydrogenase (LDH) release of stably transfected cells was determined. Expression of SP-C^I73T ^led to an overall slightly increased LDH release, suggesting some reduction in cell viability (Figure 3). Upon exposure of stable MLE-12 to pharmacological substances currently applied in ILD therapy, the cells expressing SP-C^I73T ^showed more pronounced increase in LDH release than cells expressing SP-C^WT^. Azathioprine treatment resulted in the most pronounced LDH release, while the effect of hydroxychloroquine, methylprednisolone and cyclophosphamide was significantly lower (Figure 3). This demonstrates that the expression of proSP-C^I73T ^is a stress factor that may increase cell vulnerability and susceptibility to exogenous stress. In addition, our data suggest that some substances used in the ILD therapy are a potent (azathioprine) to moderate (hydroxychloroquine) or milder (methylprednisolone and cyclophosphamide) stress factor *per se*.

### Modulation of chaperone level in cells expressing SP-C^WT ^and SP-C^I73T ^by pharmacologic substances

**Figure 4:**
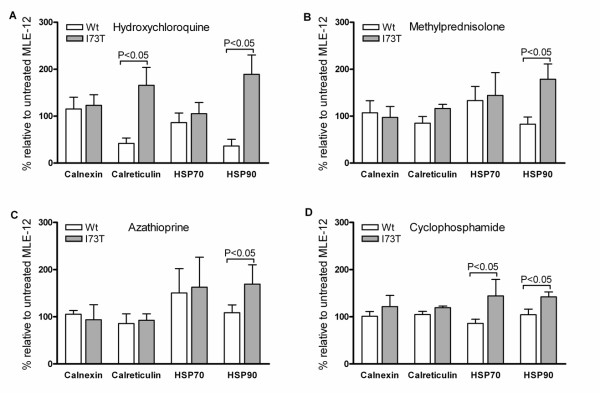
**Modulation of chaperone level in the cells expressing SP-C^WT ^and SP-C^I73T ^by pharmacologic substances**. Shown is the fold change in protein level of the chaperone proteins HSP90, HSP70, calreticulin and calnexin in MLE-12 cells expressing proSP-C^WT ^and proSP-C^I73T ^before and after their exposure to the drugs commonly applied in ILD therapy: (A) hydroxychloroquine, (B) methylprednisolone, (C) azathioprine and (D) cyclophosphamide. Only significant p-values are presented.

After demonstrating that SP-C^I73T ^expression increases cell vulnerability to pharmacological stress stimuli, we further aimed to investigate the underlying intracellular mechanisms. Chaperone proteins are involved in the folding of aberrantly processed proteins and produced by cells as a part of a cytoprotective mechanism to cope with increased intracellular stress and accumulation of misfolded proteins [29,30]. We determined a fold change in the protein level of the two heat shock proteins, HSP90 and HSP70, and two ER-resident chaperones, calreticulin and calnexin, in MLE-12 cells expressing SP-C^WT ^and SP-C^I73T^, before and after exposure to the pharmacologic substances used in ILD therapy: cyclophosphamide, azathioprine, methylprednisolone or hydroxychloroquine (Figure 4A-D, Table 1). The expression of HSP90 was increased significantly by all four pharmacologic substances tested in I73T cells compared to WT. Calreticulin was also increased in I73T mutant after the treatment with hydroxychloroquine and HSP70 expression increased after treatment with cyclophosphamide compared to WT cells. Treatment with any of the four substances did not alter the expression of calnexin between SP-C^WT ^and SP-C^I73T^-expressing cells (Figure 4). Overall, the exposure of SP-C^I73T^-expressing cells to selected pharmacologic substances increased expression of some chaperones compared to SP-C^WT ^cells, being a mechanism, which might enhance general cell folding capacity.

**Table 1:** Chaperone level in MLE-12 cells expressing mutant SP-C^I73T ^in response to drugs used in ILD therapy.

	HSP 90	Calreticulin	HSP 70	Calnexin
Cyclophosphamide	+ 30% *	+ 13% *	+ 41% *	+ 16%
Azathioprine	+36% *	+ 8%	+ 7%	- 13%
Hydroxychloroquine	+ 81% *	+ 75% *	+ 18%	+ 6%
Methylprednisolone	+ 55% *	+ 26% *	+ 8%	- 9%

Presented are the results of three independent experiments and expressed as % changes of the protein amounts in SP-C^I73T ^cells compared to SP-C^WT ^expressing cells; * denotes p-values < 0.05

### Pharmacological modulation of intracellular localization of proSP-C^WT ^and proSP-C^I73T^

**Figure 5:**
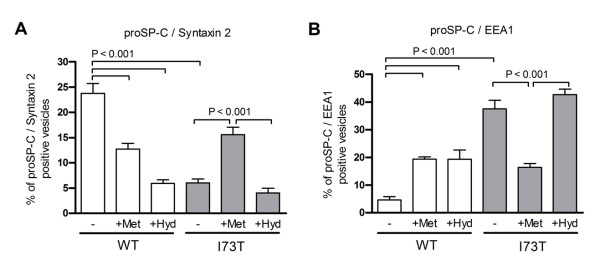
**Influence of methylprednisolone and hydroxychloroquine on intracellular localization of SP-C^WT ^or SP-C^I73T^**. Stabile MLE-12 cells expressing SP-C^WT ^or SP-C^I73T ^were incubated 24 hours with 10 μM of methylprednisolone (+Met) or hydroxychloroquine (+Hyd) and analyzed by immunofluorescence followed by quantification of colocalization or proSP-C (HA-tag) with syntaxin 2 (marker of surfactant secretory vesicles) and EEA1 (endosomal marker). Only significant p-values are shown.

Knowing that tested pharmacological substances enhance chaperone expression in cells with SP-C^I73T ^in comparison to those expressing SP-C^WT ^and that proSP-C^I73T ^forms are mislocalized to early endosomal vesicles, we investigated influence of two drugs used in ILD therapy, hydroxychloroquine and methylprednisolone, on proSP-C^WT ^and proSP-C^I73T^. We applied again syntaxin 2 and EEA1 as markers for correctly localized and mislocalized proSP-C respectively [7], in a quantitative immunofluorescence study in order to determine the percentage of proSP-C-positive vesicles that colocalized with either of the two protein markers. As shown in Figure 2, we observed high level of colocalization of proSP-C^WT ^forms with syntaxin 2 and of proSP-C^I73T ^with EEA1 (Figure 5A and 5B). Exposure to both pharmacological substances reduced the localization of proSP-C^WT ^to syntaxin 2-positive vesicles and moved it toward EEA1-positive vesicles. However, even after the drug treatment the colocalization level of WT with EEA1 remained significantly under the level detected in non-treated I73T mutant (Figure 5B). Furthermore, while hydroxychloroquine did not significantly improve mislocalization defect of the proSP-C^I73T ^forms, we observed correctional effect of methylprednisolone on localization of proSP-C^I73T^. Namely, methylprednisolone increased localization of the proSP-C^I73T ^forms to the syntaxin 2-positive (Figure 5A) vesicles and decreased their colocalization with EEA1 (Figure 5B). Nevertheless, even after the pharmacological treatment proSP-C^I73T ^never completely acquired WT localization features (Figure 5A and 5B). Our data suggest the ability of the methylprednisolone drug to partially correct mislocalization defect of proSP-C^I73T^.

### Alterations in the intracellular lipid composition and composition of secreted lipids due to expression of SP-C^I73T ^and their response to pharmacological treatment

**Figure 6:**
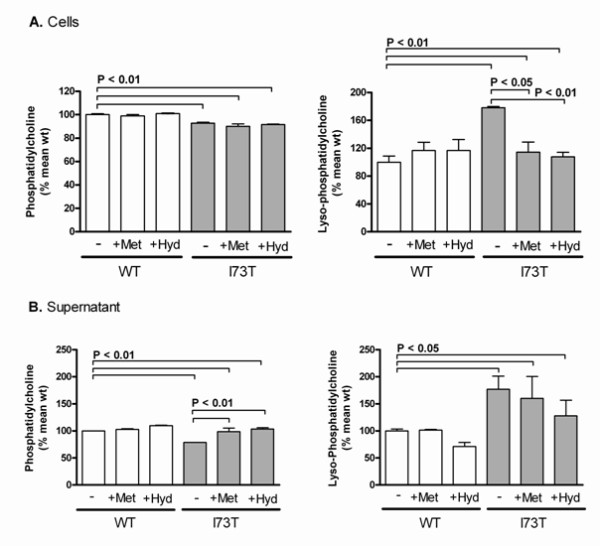
**Intracellular lipid content and lipid secretion of MLE-12 cells expressing SP-C^WT ^or SP-C^I73T^**. (A) Intracellular lipid content of cells stably expressing WT SP-C or I73T mutant were quantified by mass spectrometry. Untreated cells (-) or cells treated with 10 μM methylprednisolone (+Met) or hydroxychloroquine (+Hyd) for 24 hours prior to sample isolation. Values were calculated as % of the mean of the untreated WT values. (B) after removal of detached cells, the lipids in the cell supernatant were analysed and presented as in (A). The graphs show relative amounts of phosphatidylcholine and lyso-phosphatidylcholine. Only significant p-values are depicted.

The packaging and secretion of lung surfactant lipids is very closely linked to the expression of the hydrophobic surfactant proteins in AECII [5]. Mass spectrometric lipid analysis showed that total phospholipid amount was not changed in transfected MLE-12 cells (Table 2). However, the phospholipid composition was significantly altered: phosphatidylcholine (PC) and sphingomyelin were decreased and lyso-phosphatidylcholine (LPC) and phosphatidylethanolamine were increased in I73T mutant cells (Table 2, Figure 6). Treatment with methylprednisolone or hydroxychloroquine did not correct the loss of PC in SP-C^I73T ^expressing cells, but it did ameliorate the LPC increase (Figure 6A). Also significant changes in the pattern of the fatty acids molecular species of different phospholipid classes were measured (Table 2, Additional file 1: Supplemental Table S1), suggesting that the lipid sorting processes of the cells were also affected substantially.

**Table 2:** Phospholipid profile of transfected MLE-12 cells stably expressing mutant SP-C^I73T^.

	WT	I73T	P (Anova)
Total phospholipids (nmol/mg protein)	152.80 ± 9.6	153.10 ± 10.0	ns
Phospholipid classes (% of total PL)			
Phosphatidylcholine	57.80 ± 1.0	53.70 ± 0.8	< 0.001
Lyso-Phosphatidylcholine	0.60 ± 0.1	1.00 ± 0.8	< 0.001
Phosphatidylglycerol	0.30 ± 0.0	0.20 ± 0.1	ns
Sphingomyelin	6.20 ± 0.3	5.50 ± 0.2	< 0.05
Ceramide	1.80 ± 0.1	1.80 ± 0.1	ns
Glucosyl-Ceramide	0.10 ± 0.0	0.10 ± 0.0	ns
Phosphatidylethanolamine	11.20 ± 0.5	14.70 ± 0.8	< 0.001
Phosphatidylserine	6.50 ± 0.1	6.60 ± 0.3	ns

Data are means of three independent experiments each performed in duplicate. P-values are given.

The phospholipid secretion by MLE-12 cells was assessed in the supernatant. Similar as in the intracellular lipid pattern, PC was decreased by 27% and LPC was increased by 57% in cells expressing SP-C^I73T ^(Figure 6B), with no changes detected for other phospholipids (data not shown). Interestingly, the treatment with methylprednisolone or hydroxychloroquine ameliorated the reduction of PC, but had no effect on LPC. Our data suggest that the expression of SP-C^I73T ^affected the lipid composition of AECII and alveolar pulmonary surfactant profoundly. The major surfactant phospholipid PC was reduced with a concomitant increase in LPC, suggesting increased activity of phospholipases. Treatment with methylprednisolone or hydroxychloroquine corrected to some extent these alterations back toward the WT level.

### MLE-12 cells expressing SP-C^I73T ^secrete soluble factors that stimulate surface expression of CCR2 and CXCR1 on CD4+ lymphocytes and CXCR1 on neutrophils

**Figure 7:**
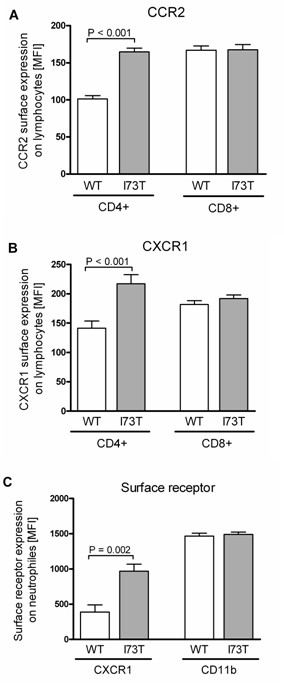
**Surface receptor expression on human lymphocytes and neutrophils**. Neutrophils and lymphocytes were isolated from the whole blood of different human donors and incubated with 7-fold concentrated supernatants obtained from MLE-12 cells expressing SP-C^WT ^or SP-C^I73T ^prior to flow cytometry analysis. Non-concentrated supernatants gave the same results, although less pronounced with a clear concentration dependency of the effects (data not shown). The receptor levels on the surface of lymphocytes after incubation with antibodies directed against (A) CCR2 or (B) CXCR1 are shown and expressed as mean fluorescence intensity (MFI). Another second marker-specific antibody was applied to distinguish between CD4+ and CD8+ lymphocytes. (C) The levels of CXCR1 and CD11b on isolated neutrophils. Significant changes are depicted with the corresponding p-values.

Injury of the lung epithelial cell caused by endogenous and exogenous stress may be communicated to the surrounding immune cells, in particular to the pulmonary leukocytes, leading to inflammation and cell remodeling. Chemokine receptors recruit leukocytes to the alveolar site of inflammation and orchestrate local immune responses [33,34]. In the previous studies we demonstrated that, among a plethora of chemokine receptors involved in this network, specifically CCR2 on lymphocytes and CXCR1 on neutrophils, modulate pulmonary immunity in human inflammatory lung diseases [35,36]. Therefore, we examined whether cells expressing SP-C^I73T ^stimulated the expression of CCR2 on lymphocytes and CXCR1 on neutrophils by incubating isolated neutrophils or lymphocytes with 7-fold concentrated supernatants of MLE-12 cells expressing WT or I73T SP-C. As a result, CD8+ lymphocytes did not show a difference between WT and I73T mutant, however CD4+ lymphocytes showed an increased level of surface receptor CCR2 expression in response to the supernatant of proSP-C^I73T ^expressing cells (Figure 7A). We observed the same pattern with CXCR1, which was increased on CD4+ lymphocytes after incubation with the mutant cell supernatant, but was unaltered on CD8+ lymphocytes (Figure 7B). We further analyzed the surface receptor expression on neutrophils. The supernatant of SP-C^I73T ^expressing cells increased the level of CXCR1 expression on neutrophils, but did not affect CD11b levels (Figure 7C). Non-concentrated supernatants gave the same results, although less pronounced and a clear concentration dependency of the effects was observed (data not shown). This suggests that SP-C^I73T^-expressing MLE-12 cells were able to modulate the surface receptor expression on the cells of immune system through the secretion of soluble factors into the medium.

## Discussion

Mutations in the *SFTPC *gene are a known cause of surfactant deficiency and very variable genetic ILD in children and adults. We investigated the intracellular disturbances and intercellular signaling of MLE-12 cells expressing SP-C^I73T ^and the ability of pharmaceutical drugs used in ILD therapy to modulate some of the cellular consequences of SP-C deficiency caused by this mutation. MLE-12 cells were chosen as a model system since they contain structures, which resemble lamellar bodies seen in AECII [37]. The presence of lamellar body-like structures in the cells used was confirmed by electron microscopy (data not shown). Here we named the organelles detectable as LAMP3-positive vesicles, lamellar body-like structures.

A potential limitation of the study is that our system corresponds rather to a homozygous than to a heterozygous *SFTPC *mutation where one WT copy is still present. Although endogenous SP-C is expressed in the MLE-12 cells [38], expression of exogenous SP-C from the CMV promoter present on the plasmid vector is likely higher. However, all known patients with SP-C mutations are heterozygous, expressing one copy of the wild type gene. Thus, the experimental model reflects the *in vivo *condition. In addition, in contrast to SP-C^Δexon4 ^or SP-C^L188Q^, I73T is a non-BRICHOS domain mutation and does not lead to the conformational disease observed for BRICHOS domain mutations where the mutant allele acts in a dominant-negative way [13]. Consequently, I73T still allows for the production of mature SP-C *in vivo *[17].

Stable transfection of MLE-12 cells with SP-C^WT ^or SP-C^I73T ^led to the intracellular accumulation of proSP-C^I73T ^processing intermediates which were not found in cells with proSP-C^WT^, but corresponded well to species in the BAL fluid of patients with this mutation [17] (Figure 1). The first step in proSP-C processing is a cleavage at the C-terminal end [2]. Using an EGFP-tag fused to the C-terminus of proSP-C showed no difference in processing intermediates of proSP-C^WT ^and proSP-C^I73T ^(Figure 1, right). This means that (a) the first cleavage step happening at C-terminus is not influenced by this mutation and (b) the mutation does not interfere with the export from the ER and Golgi, because this cleavage occurs after trafficking through these compartments [2,3]. In addition, immunofluorescence assays showed neither proSP-C^WT ^nor proSP-C^I73T ^retention in the ER compartment (Figure 2), supporting the conclusions made from the immunoblots. To examine the processing following the first C-terminal cleavage, we applied N-terminal protein tags. Dominant proSP-C^WT ^intermediates, that were also identified for proSP-C^I73T^, were the species after the first C-terminal cleavage (Figure 1, bands #2 and #6), and the species before the first N-terminal cleavage (Figure 1, band #8). The primary full-length translation product (Figure 1, bands #1 and #5) was only faintly detectable for proSP-C^WT^. Expression of proSP-C in this model is under control of a CMV-promoter, not the native SP-C promoter. It is therefore unlikely that a feedback mechanism is responsible for a higher expression of proSP-C^I73T ^intermediates. It is more likely that the I73T mutation slows down the processing and/or trafficking of the mutant proSP-C, leading to accumulation of incompletely processed proSP-C. It is not known how this mutation affects the folding of proSP-C, but subtle changes in conformation may also be responsible for the abundance of another processing intermediate, of size ~17 kDa (Figure 1, band #3 and #7). This intermediate can be found in the BAL fluid of patients with the I73T mutation, suggesting that this proSP-C form is being secreted from AECII along with the mature SP-C that is produced by AECII regardless of the presence of the I73T mutation [17].

Immunofluorescence assay of stably transfected MLE-12 showed that proSP-C^I73T ^colocalized often with EEA1 positive vesicles (Figure 2A), confirming our previous report (12). Early endosomes generally contain material that is taken up by endocytosis and is either recycled or routed for degradation [32]. Up to 80% of used lung surfactant is known to be reinternalized by AECII from alveolar space [5]. Although immunofluorescence does not allow the distinction between different EGFP-positive species depicted in Figure 1B, we speculate that the proSP-C^I73T ^species in the EEA1 positive compartment might be primarily the additional preprotein species accumulating in the I73T mutant. They are secreted only by the AECII with the I73T mutation and might be reinternalized as well. On the other hand, proSP-C^WT^, but rarely proSP-C^I73T^, colocalized with syntaxin 2, a SNARE protein involved in the secretion of lung surfactant, found in the plasma membrane and lamellar bodies of AECII (Figure 2B). Interestingly, our data propose the influence of hydroxychloroquine and methylprednisolone on localization and routing of proSP-C^WT ^moving it toward early endosomal vesicles. On the other hand, methylprednisolone showed the capacity to partially correct the mislocalization/routing defect of proSP-C^I73T ^(Figure 5).

The expression of mutated proteins frequently results in elevated cell stress. This has been shown for the BRICHOS domain SP-C mutations L188Q and Δexon4 [14,15]. We found that the constitutive expression of SP-C^I73T ^moderately increased cell lethality under normal growth conditions (Figure 3A), maybe as a result of the ability of the cellular system to adapt to the presence of stress, as reported in [39]. The additional exogenous stress, imposed in our experiments by exposure to pharmaceuticals used in ILD therapy, might shift this balance out of the tolerable range. Treatment of the cells with azathioprine drug almost doubled the number of dying I73T mutant cells compared to WT. This aggravation was much less pronounced in the presence of methylprednisolone, hydroxychloroquine or cyclophosphamide.

Intracellular stress is in part handled by endogenous chaperones. Still without pharmacological boost, such cytoprotective mechanisms may not always be sufficient to normalize the cell function and maintain production of the bioactive surfactant with a normal lipid/protein composition. We determined the change in expression of the four important chaperones under the influence of the same ILD drugs. We found that the influence of azathioprine on the chaperones was almost the same in proSP-C^WT^-and proSP-C^I73T^-expressing cells, leaving no protection for additional stress, being a potent stress factor *per se *(Figure 4A-D). In contrast, hydroxychloroquine treatment led to an 81% increase in HSP90, and 75% increase in calreticulin expression in I73T mutant cells over WT cells (Table 1), thereby possibly protecting the cells against the additional stress and enhancing the ER folding capacity. HSP90 seemed to be targeted by all tested pharmaceuticals, while calnexin levels were refractory to stimulation (Figure 4). Treatment with the four drugs did not change the pattern of the proSP-C processing bands observed in the immunoblots in Figure 1A (data not shown).

The lipid composition of the stable MLE-12 cells was similar to that previously described in human foetal AECII, especially with regard to PC composition [40]. In the SP-C^I73T ^expressing cells we found a pronounced drop of total cellular PC, whereas LPC was increased (Figure 6A, additional file 1: supplemental Table S1). It is known that PC is degraded to LPC by an intrinsic phospholipase A2-like activity, and that LPC is toxic to various cells [41]. Increased LPC may therefore be a result of increased phospholipase activity due to the presence of mutated SP-C. SP-C dysfunction may also lead to a diminished activity of acyltransferases which reacetylate LPC. LPC is a known inhibitor of the lung surfactant activity and has the ability to penetrate directly into interfacial films to impair lowering of the alveolar surface tension during dynamic compression [42,43]. Elevated LPC levels in the SP-C^I73T^-expressing cells could also explain the heightened sensitivity towards exogenous stress described above. Generation of LPC cannot account for the decrease of PC mass in SP-C^I73T ^expressing cells, but additional factors, which directly interfere with the synthesis and packaging of PC, must also be responsible. This is in line with the observed grossly altered pattern of the fatty acid species of the different phospholipid classes, including PC in SP-C^I73T ^cells (Table 2, additional file 1: supplemental Table S1).

AECII secrete the surfactant phospholipids into the alveolar space where it lowers surface tension. Among phospholipids secreted by the I73T mutants PC was again decreased by 27% and LPC was increased by 57%, compatible with a reduced surfactant function [40,43]. Treatment with methylprednisolone or hydroxychloroquine ameliorated the increase in intracellular and secreted LPC and decrease in secreted PC, but did not completely correct it (Figure 6A, B). The capacity of the treatment with methylprednisolone and hydroxychloroquine to correct the lipid disturbances caused by I73T mutation (Figure 6B) represent one of the mechanisms by which these treatments are empirically helpful in some patients with I73T mutations (own unpublished results, [19]).

Lastly, the index patient with the I73T mutation in our previous study displayed a mild interstitial chronic inflammation and most of the infiltrated leukocytes were CD3+ and CD4+ T-lymphocytes [17]. We found that cells with the I73T mutation released soluble factors into the medium that increase surface expression of CCR2 and CXCR1 on CD4+ lymphocytes and CXCR1 on neutrophiles (Figure 7A-C). When activated, the high affinity IL-8 receptor CXCR1 mediates antibacterial killing capacity [36,44]. Increases in surface expression levels of CCR2 and CXCR1, respectively, might have the potential to modulate the pulmonary immune response with regard to antibacterial (CXCR1) and profibrotic (CCR2) responses [36,45]. However, the soluble factors involved in the induction of chemokine receptor expression as well as the functional consequences of this phenomenon remain to be addressed in future studies.

## Conclusions

We showed impaired proSP-C processing, altered cellular stress tolerance and unfavorable changes of the surfactant lipid composition in a murine AECII model cell line. Some of the demonstrated cellular aspects behind the disease could be modulated with drugs used in the therapy of ILD patients, thereby giving insight into their potential therapeutic mechanism on a cellular level. We also demonstrated that AECII with I73T mutation could signal to the surrounding cells of the immune system through secretion of soluble factors. Therefore, our study adds to understanding of the effects that *SFTPC *mutations impose on (pro)SP-C and AECII cell biology and pave the way for a more precise pharmacological targeting in patients with SP-C deficiency.

## Methods

### Plasmid vectors

Eukaryotic expression vectors containing the full human *SFTPC *gene fused to either EGFP-tag (pEGFP-N1/hSP-C^1-197 ^and pEGFP-C1/hSP-C^1-197 ^to obtain proSP-C with EGFP fused to the C- or N-terminus respectively) or hemagglutinin (HA)-tag (proSP-C with N-terminal HA-tag) were obtained as previously described [12]. I73T point mutation was introduced into the wild-type (WT) *SFTPC *gene in these vectors using the QuikChange site-directed mutagenesis kit (Stratagene, La Jolla, USA) and the following primers: I73T_forward: 5'-GGT TCT GGA GAT GAG CAC TGG GGC GC-3', I73T_reverse: 5'-GCG CCC CAG TGC TCA TCT CCA GAA CC-3', following the recommended protocol. The successful mutagenesis was confirmed by DNA sequencing.

### MLE-12 cell lines and transfection

The mouse MLE-12 lung epithelial cell line (CRL-2119) [38] was obtained from the American Type Culture Collection (ATCC) and maintained in RPMI medium supplemented with 10% FBS. Cells were transfected using FuGene 6 (Roche, Penzberg, Germany) according to the manufacturer's protocol. Stable transfection of MLE-12 cells with pcDNA3/HA-hSP-C^1-197 ^and pcDNA3/HA-hSP-C^I73T ^vectors was obtained by selecting transfected cells in the presence of 600 μg/ml G418 in RPMI medium for four weeks. For drug exposure experiments stable cells were grown 24 hours in the presence of 10 μM of cyclophosphamide, azathioprine, methylprednisolone or hydroxychloroquine.

### Immunoblotting

Total cell proteins were obtained by lysing the cells in lysis buffer (PBS, 20 mM EDTA, 1% v/v Elugent (Calbiochem, Bad Soden, Germany), protease inhibitor (Complete; Roche, Manheim, Germany). For immunoblotting 30 μg protein were separated under reducing conditions using 10% NuPage Bis-Tris (Invitrogen, Karlsruhe, Germany) and transferred to a PVDF membrane. The following primary antibodies were used: monoclonal rat anti-HA-tag (1:1000; Roche), monoclonal mouse anti-GFP (1:500; Clontech, Heidelberg, Germany) and polyclonal goat anti-calnexin (1:500), polyclonal goat anti-calreticulin (1:500), monoclonal mouse anti-HSP90α/β, polyclonal goat anti-HSP70 (1:1000) and monoclonal anti-β-actin HRP conjugate (1:10000) (all from Santa Cruz Biotechnology, Santa Cruz, CA). Signal was detected using chemiluminiscent labeling with Amersham ECL Detection Reagents (GE Healthcare), densitometrically quantified and normalized to the β-actin signal.

### Immunofluorescence

24 hours after transfection cells grown on coverslips were fixed with 4% paraformaldehyde, permeabilised with 10% Triton X-100, blocked 30 min in PBS with 5% FBS. The following primary antibodies were used and all in 1:200 dilution: polyclonal rabbit anti-mouse LAMP3 (Santa Cruz), monoclonal mouse anti-human CD63/LAMP3 (Chemicon, Schwalbach, Germany), polyclonal rabbit anti-EEA1 (Acris Antibodies, Herford, Germany), monoclonal mouse anti-ubiquitin (Biomol, Hamburg, Germany) and polyclonal rabbit anti-syntaxin 2 (Synaptic Systems, Berlin, Germany). Species specific Alexa Fluor 488 or Alexa Fluor 555 secondary antibodies (Invitrogen) were used at 1:200. Samples were mounted and Alexa Fluor or GFP fluorescence was examined with Axiovert 135 fluorescent microscope and evaluated with AxioVision 4.7.1 software (Carl Zeiss, Jena, Germany). For semi-quantitative assessment of colocalization, high magnification confocal microscope images were used. On 14 to 27 different coverslips at least 100 vesicles stained for SP-C and/or syntaxin 2 were counted in a blinded fashion and the percentage of vesicles showing staining for both markers was calculated. Similarly, the percentage of vesicles stained for SP-C and EEA-1 was calculated.

### Lactate dehydrogenase (LDH) assay

LDH activity in cell lysates and culture supernatants was determined by the method of Decker and Lohmann-Matthes [46]. Briefly, 100 μl of samples were mixed with 30 μl dye solution (18 mg/ml L-lactate, 1 mg/ml iodonitrotetrazolium in PBS). After adding 15 μl of the catalyst (3 mg/ml NAD^+^, 2.3 mg/ml diaphorase, 0.03% BSA, 1.2% sucrose in PBS), absorbance at 492 nm was determined at one minute intervals for 15 minutes at 37°C. Absolute LDH activity was calculated from a standard curve, using purified LDH (Sigma, Munich, Germany). The lower limit of detection was 20 Units/L; the assay was linear to 2500 Units/L.

### Mass spectrometric lipid analysis

For lipid analysis cells grown in Petri dishes were harvested by scraping off in 2 mL PBS supplemented with protease inhibitor (Complete, Roche). The cell suspension was sonicated (four strokes, 10 seconds; Branson Digital Sonifier S450D). Lipid classes and subspecies were determined by electrospray ionization tandem mass spectrometry (ESI-MS/MS) using direct flow injection analysis, as described previously [47,48]. Cells were extracted according to the Bligh and Dyer method in the presence of non-naturally occurring lipid species used as internal standards [49]. A precursor ion scan of *m/z *184 specific for phosphocholine containing lipids was used for phosphatidylcholine (PC), sphingomyelin (SPM) [48] and lysophosphatidylcholine (LPC) [47]. Neutral loss scans of *m/z *141 and *m/z *185 were used for phosphatidylethanolamine (PE) and phosphatidylserine (PS), respectively [50]. Phosphatidylglycerol (PG) was analyzed using a neutral loss scan of *m/z *189 of ammonium adduct ions [51]. Ceramide and glucosylceramide were analyzed as previously described [52] using N-heptadecanoyl-sphingosine as internal standard. Quantification was achieved by calibration lines generated by addition of naturally occurring lipid species to pooled cell homogenate. All lipid classes were quantified with internal standards belonging to the same lipid class, except SM (PC internal standards). Each lipid class was calibrated with a variety of species covering chain lengths and number of double bonds of naturally occurring species. Correction of isotopic overlap of lipid species and data analysis was performed by self-programmed Excel macros for all lipid classes according to the described principles [48].

### Flow cytometry

Human lymphocytes and neutrophils were isolated from whole blood using LeucoSep (Greiner Bio-One, Solingen-Wald, Germany) and Ficoll-Isopaque gradient density isolation method (GE Healthcare) according to the manufacturer's instructions. Cells were incubated for 6 hours (neutrophils) or 24 hours (lymphocytes) at 37°C with supernatants of MLE-12 cells expressing wild-type or mutant proSP-C. Cell-free supernatants were collected after 48 hours of growth and concentrated 7-fold, using Microsep 1 k centrifugal concentrators (Millipore, Schwalbach, Germany). Cells were analyzed by four-colour flow cytometry (FACSCalibur; BD Pharmingen, Heidelberg, Germany) as described previously [35]. The following antibodies were used: PE-conjugated mouse anti-human CCR2-B (R&D Systems, Minneapolis, USA), FITC labeled anti-human CD8, FITC labeled anti-human CD4, PE-conjugated mouse anti-human CD11b/Mac-1, PE-conjugated mouse anti-human CD181 (CXCR1, IL-8RA) (all BD Pharmingen). Results are presented as mean fluorescence intensity (MFI) after subtracting background binding provided by non-specific isotypes. Calculations were performed with CellQuest analysis software (BD Pharmingen).

### Statistical methods

Since the data was distributed non-Gaussian, non-parametric tests were used for comparison of two unpaired groups (Mann Whitney test). The results are given as means ± standard error (SE) of the individual number of different subjects, each individual value representing the mean of 3-4 determinations or as indicated. For lipid analysis the results are presented as means with standard deviation and comparisons were made by ANOVA followed by Tukey's post hoc multi comparisons test. For correlations, Spearman's non-parametric test was used. P-values of less than 0.05 were considered statistically significant.

## List of abbreviations

SP-C: surfactant protein C; *SFTPC*: surfactant protein C gen; SP-B: surfactant protein B; ILD: interstitial lung disease; BAL: bronchial lavage; AECII: alveolar type II cells; SNARE: SNAP receptors; SNAP-23: soluble NSF attachment protein 23; EGFP: enhanced green fluorescent protein; HA: hemagglutinin; LAMP3: lysomal-associated membrane protein 3; EEA1: early endosomal antigen 1; HRP: horseradish peroxidase; LDH: lactate dehydrogenase; ER: endoplasmic reticulum; HSP70/90: heath shock protein 70/90; PC: phosphatidylcholine; LPC: lysophosphatidylcholine; PE^1^: phosphatidylethanolamine; PS: phosphatidylserine; SPM: sphingomyelin; PG: phosphatidylglycerol; CXCR1: CXC chemokine receptor; CCR2: chemokine C-C motif receptor 2; FITC: fluorescein isothiocyanate; PE^2^: phycoerythrin; MFI: mean fluorescence intensity; CYC: cyclophosphamide; AZA: azathioprine; MET: methylprednisolone; HYD: hydroxychloroquine.

## Authors' contributions

MG, MW, CS and SK conceived and designed the study as well as analyzed and interpreted the data. CS, MW, SK, TT performed the experiments with exception of MS lipid analyses, performed by GL and GS, and flow cytometry analyses, performed by AH and DH. MW, SK and MG wrote the paper. SM and MFB generated and provided the necessary wild type vectors and participated in drafting and reviewing the manuscript. All authors read and approved the final manuscript.

## Authors' information

This paper contains parts of the doctoral thesis of Christiane Sparr. Until 2009 Sunčana Kern published as Sunčana Moslavac.

## Supplementary Material

Additional file 1 **Table S1. Phospholipid classes and molecular species profile of transfected MLE-12 cells stably expressing *SFTPC *I73T mutation**. Data are means and standard deviation of three independent experiments, each performed in duplicate. The mutant (I73T) and wild type (WT) were compared by ANOVA followed by Tukeys multiple comparison test. P-values are shown. Click here for file
