# Supplementary material for: A non-BRICHOS surfactant protein c mutation disrupts epithelial cell function and intercellular signaling
Source: BMC Cell Biol. 2010 Nov 20;11:88. doi: 10.1186/1471-2121-11-88 (PMC2994813; doi:10.1186/1471-2121-11-88)
Supplement: Additional file 1 — Table S1. Phospholipid classes and molecular species profile of transfected MLE-12 cells stably expressing SFTPC I73T mutation. Data are means and standard deviation of three independent experiments, each performed in duplicate. The mutant (I73T) and wild type (WT) were compared by ANOVA followed by Tukeys multiple comparison test. P-values are shown. [file 1471-2121-11-88-S1.PDF]

Supplemental material

Supplemental Table 1.

	WT	*I73T*	*P (Anova)*
Total phospholipids (nmol/mg protein)	*152.80 ± 9.6*	*153.10 ± 10.0*	*> 0.05*
Phosphatidylcholine (% of total PL)	*57.80 ± 1.0*	*53.70 ± 0.8*	*< 0.001*
PC 32:1 (% of PC)	22.92 ± 2.4	22.21 ± 2.4	> 0.05
PC 34:1 (% of PC)	14.65 ± 0.3	14.54 ± 0.6	> 0.05
PC O 32:1 (% of PC)	8.7 ± 0.1	7.1 ± 0.1	< 0.001
PC O 34:1 (% of PC)	5.6 ± 0.5	4.6 ± 0.6	< 0.01
PC O 32:0 (% of PC)	5.3 ± 0.9	3.9 ± 0.6	< 0.05
PC 34:2 (% of PC)	7.30 ± 0.1	7.82 ± 0.5	> 0.05
PC 30:0 (% of PC)	5.69 ± 0.2	7.16 ± 0.1	< 0.001
PC 36:2 (% of PC)	5.19 ± 0.2	6.01 ± 0.1	< 0.01
PC 32:0 (% of PC)	5.18 ± 0.8	4.61 ± 0.6	> 0.05
PC 32:2 (% of PC)	3.30 ± 0.2	3.81 ± 0.6	> 0.05
PC 30:1 (% of PC)	2.65 ± 0.4	3.54 ± 0.7	> 0.05
PC 36:1 (% of PC)	1.51 ± 0.0	1.04 ± 0.1	< 0.001
Lyso-Phosphatidylcholine (% of total PL)	*0.60 ± 0.1*	*1.00 ± 0.8*	*< 0.001*
LPC 16:0 (% of LPC)	53.4 ± 2.2	51.37 ± 1.0	> 0.05
LPC 18:1 (% of LPC)	17.73 ± 0.5	20.03 ± 0.6	< 0.001
LPC 16:1 (% of LPC)	10.50 ± 0.8	12.73 ± 2.0	> 0.05
LPC 18:0 (% of LPC)	8.53 ± 1.0	6.67 ± 1.3	> 0.05
LPC 15:0 (% of LPC)	3.83 ± 0.4	2.93 ± 0.1	> 0.05
Phosphatidylglycerol (% of total PL)	*0.30 ± 0.0*	*0.20 ± 0.1*	*> 0.05*
PG 34:1 (% of PG)	71.47 ± 0.7	64.22 ± 0.8	< 0.001
PG 34:2 (% of PG)	6.50 ± 0.9	9.49 ± 0.3	< 0.001

PG 36:2 (% of PG)	5.87 ± 0.7	9.89 ± 0.5	< 0.001
PG 32:1 (% of PG)	5.57 ± 0.5	4.96 ± 0.6	> 0.05
PG 36:1 (% of PG)	5.27 ± 0.5	5.14 ± 0.1	> 0.05
PG 32:2 (% of PG)	1.36 ± 0.0	1.56 ± 0.2	> 0.05
PG 30:0 (% of PG)	1.56 ± 0.3	1.54 ± 0.1	> 0.05
PG 36:3 (% of PG)	0.97 ± 0.2	1.57 ± 0.4	> 0.05
PG 32:0 (% of PG)	1.26 ± 0.2	0.87 ± 0.1	> 0.05
Sphingomyelin (% of total PL)	*6.20 ± 0.3*	*5.50 ± 0.2*	*< 0.05*
SPM 16:0 (% of SPM)	56.32 ± 1.1	44.11 ± 0.2	< 0.001
SPM 24:1 (% of SPM)	14.43 ± 0.6	19.06 ± 0.3	< 0.001
SPM 16:1 (% of SPM)	7.47 ± 0.3	7.10 ± 0.2	> 0.05
SPM 24:0 (% of SPM)	6.20 ± 0.1	4.81 ± 0.3	< 0.001
SPM 24:2 (% of SPM)	3.16 ± 0.3	6.02 ± 0.3	< 0.001
SPM 22:1 (% of SPM)	2.02 ± 0.1	6.10 ± 0.3	< 0.001
SPM 22:0 (% of SPM)	1.98 ± 0.3	3.10 ± 0.2	< 0.01
Ceramide &	*1.80 ± 0.1*	*1.80 ± 0.1*	*> 0.05*
Glucosyl-Ceramide (% of total PL)	*0.10 ± 0.0*	*0.10 ± 0.0*	*> 0.05*
Cer 16:0 (% of Cer and GluCer)	58.73 ± 0.4	49.43 ± 0.5	< 0.001
Cer 24:1 (% of Cer and GluCer)	21.53 ± 0.8	30.23 ± 0.4	< 0.001
Cer 24:0 (% of Cer and GluCer)	13.67 ± 0.4	11.20 ± 0.2	< 0.001
Cer 22:0 (% of Cer and GluCer)	4.27 ± 0.2	6.67 ± 0.1	< 0.001
Glu Cer 16:0 (% of Cer and GluCer)	3.20 ± 0.3	3.03 ± 0.4	> 0.05
Glu Cer 24:1 (% of Cer and GluCer)	1.77 ± 0.1	3.00 ± 0.1	< 0.01
Phosphatidylethanolamine (% of total PL)	*11.20 ± 0.5*	*14.70 ± 0.8*	*< 0.001*
PE 34:1 (% of PE)	25.91 ± 0.2	24.02 ± 0.7	< 0.05
PE 34:2 (% of PE)	24.49 ± 0.2	25.24 ± 1.0	> 0.05

PE 32:1 (% of PE)	17.38 ± 1.3	13.68 ± 1.7	< 0.05
PE 36:2 (% of PE)	11.31 ± 0.5	14.12 ± 0.7	< 0.01
PE 32:2 (% of PE)	6.83 ± 0.2	6.07 ± 0.7	> 0.05
PE 36:1 (% of PE)	4.42 ± 0.2	3.90 ± 0.4	> 0.05
PE 34:3 (% of PE)	1.37 ± 0.1	1.81 ± 0.1	< 0.001
PE 32:0 (% of PE)	0.63 ± 0.1	0.51 ± 0.1	> 0.05
Phosphatidylserine (% of total PL)	*6.50 ± 0.1*	*6.60 ± 0.3*	*> 0.05*
PS 36:1 (% of PS)	26.97 ± 0.3	24.05 ± 0.9	< 0.05
PS 34:1 (% of PS)	22.61 ± 1.3	18.40 ± 1.0	< 0.01
PS 36:2 (% of PS)	15.51 ± 0.3	18.62 ± 0.1	< 0.001
PS 32:1 (% of PS)	6.07 ± 0.2	5.79 ± 0.5	> 0.05
PS 34:2 (% of PS)	5.42 ± 0.5	5.19 ± 0.8	> 0.05
PS 40:6 (% of PS)	4.40 ± 0.3	3.15 ± 0.1	< 0.001
PS 38:2 (% of PS)	4.09 ± 0.3	6.52 ± 0.1	< 0.001
PS 40:5 (% of PS)	2.99 ± 0.1	2.76 ± 0.2	> 0.05
PS 40:7 (% of PS)	2.33 ± 0.3	2.41 ± 0.0	> 0.05
PS 38:3 (% of PS)	2.46 ± 0.2	3.65 ± 0.4	< 0.001

Phospholipid classes and molecular species profile of transfected MLE-12 cells stably expressing *SFTPC* I73T mutation. Data are means and standard deviation of three independent experiments, each performed in duplicate. The mutant (I73T) and wild type (WT) were compared by ANOVA followed by Tukeys multiple comparison test. P-values are shown.
